# The Conditional Effect of Income Inequality on Obesity: A Cross-National Comparative Analysis

**DOI:** 10.1177/00469580261444418

**Published:** 2026-04-21

**Authors:** Qian Hu, Wei Zhong, Rong Hu

**Affiliations:** 1 Finance and Economics College, Jimei University, Xiamen, China; 2 Department of Sociology, University of California San Diego, USA; 3 School of Sociology and Anthropology, Xiamen University, China

**Keywords:** obesity, income inequality, social determinants of health

## Abstract

Research on income inequality and obesity shows inconsistent results. This study tests if this relationship depends on a country’s existing inequality level. We analyzed individual-level data (N = 22 938) from the 2011 and 2021 International Social Survey Programme. The sample included 10 affluent nations, divided into low-Gini and high-Gini groups. We used two-way fixed-effects linear probability models to estimate how changes in the Gini coefficient affect obesity risk. The effect of inequality on obesity is conditional. In low-Gini countries, a one-unit increase in the Gini coefficient was associated with a 2.0 percentage point higher probability of obesity (*P* < .01). However, we found no significant association in high-Gini countries. The health impacts of rising inequality are most severe when they disrupt established egalitarian norms. We suggest “ideological buffering” may explain the lack of effect in highly unequal societies, reflecting how profound stratification is often normalized and justified.

## Introduction

The prevalence of obesity has escalated into a global public health crisis, with rates continuing to climb in both developed and developing nations.^1^ While individual-level determinants such as genetics, diet and physical activity are well-documented, a substantial body of sociological research has shifted focus toward the broader social determinants of health that shape these behaviors and outcomes at the population level.^2,3^ Among these, socioeconomic status (SES) is a robust predictor of health, with a steep inverse gradient between SES and obesity in high-income countries.^4,5^

Beyond individual socioeconomic position, the contextual effect of a society’s overall level of income inequality has emerged as a critical area of inquiry in social epidemiology and health sociology.^6^ The dominant “inequality hypothesis” posits that national income inequality acts as a psychosocial stressor. This stressor erodes social cohesion and generates status anxiety, which ultimately contributes to adverse health outcomes.^7,8^ A parallel neo-materialist explanation suggests that inequality leads to underinvestment in public goods, creating more obesogenic environments.^9^ Distinct from this, the economic insecurity hypothesis posits that the mechanism linking economic conditions to health is the stress produced by widespread economic uncertainty and insecurity. This condition is driven by rapid market changes and the erosion of social safety nets, rather than by static inequality itself. This insecurity is thought to foster myopia and impatience. Consequently, individuals may prioritize immediate gratification, such as consuming cheap, energy-dense foods, over long-term health investments.^10^

Despite the theoretical appeal of these frameworks, empirical evidence for a universal association between national inequality and obesity remains inconsistent. Some cross-national studies have identified a significant positive relationship,^11^ while others have found weak or null effects.^12,13^ This heterogeneity may stem from several sources. Health research often treats affluent countries of the Global North as a homogeneous group. These nations are typically contrasted primarily with the Global South. However, this approach masks considerable diversity in socio-political models and inequality regimes within the Global North. These structural differences may be amplifying over time. Furthermore, inconsistencies may also arise from methodological limitations, as many foundational studies relied on aggregate-level ecological data, which cannot disentangle compositional effects from true contextual effects. The prevailing assumption that the impact of rising inequality is uniform across these varied societal contexts thus remains undertheorized and empirically untested.

This study addresses these complexities through a comparative approach that acknowledges the heterogeneity within affluent nations, leveraging the strengths of individual-level data from the International Social Survey Programme (ISSP) for the years 2011 and 2021. This design allows for robust control of individual socioeconomic factors, thereby mitigating the risk of ecological fallacy. We build our central argument on the premise that the societal context of income inequality matters profoundly. In societies characterized by historically low inequality and strong egalitarian norms (eg, Nordic and some continental European welfare states), social cohesion and trust are typically high. In such contexts, a rapid increase in income disparity is not merely a statistical shift. Instead, it acts as a salient social disruption that can violate deeply ingrained norms of fairness. This disruption makes social comparisons more acute and heightens status anxiety. Conversely, in societies where high income inequality is an entrenched feature of the social structure (eg, liberal market economies), the psychosocial pathways linking inequality to health may already be operating at a high level, and individuals may be more habituated to a long social ladder and status competition. We therefore hypothesize that in societies with historically low inequality, an increase in income disparity represents a more salient social shock, triggering the pathways of status anxiety and economic insecurity more acutely. Conversely, in societies where high inequality is already entrenched, a further increase may not produce a statistically distinguishable effect. To test this, we analyze 2 distinct clusters of nations: a high-Gini group (Australia, United States, Israel, Italy, Russia) and a low-Gini group (Czechia, Finland, Norway, Slovakia, Slovenia). Through this stratified analysis, this paper aims to provide a more nuanced understanding of the contextual dynamics shaping the relationship between income inequality and population health.

## Methods

### Data and Sample

This study is a cross-national comparative observational study utilizing data from the “Health and Health Care I & II – Cumulation” dataset from the ISSP, combining waves from 2011 and 2021.^14^ The ISSP is an international collaborative program conducting annual surveys on topics relevant to the social sciences, administered to nationally representative samples of adults aged 18 and over. The topical focus on health provides rich data on individual health outcomes and sociodemographic characteristics. This study followed the Strengthening the Reporting of Observational Studies in Epidemiology (STROBE) reporting guidelines (see Supplemental Material).

The complete ISSP cumulation dataset encompasses 22 countries and regions. Because our theoretical framework explicitly focuses on affluent nations of the Global North, developing economies with high income inequality (eg, South Africa, the Philippines and China) were excluded from the sampling frame. From the remaining eligible countries, our analytical strategy involved a purposive selection of 10 nations to construct 2 distinct groups representing divergent inequality regimes. The “high-Gini” group includes Australia, the United States, Israel, Italy and Russia, while the “low-Gini” group comprises Czechia, Finland, Norway, Slovakia, and Slovenia. These specific nations were selected because they consistently ranked as the 5 highest and 5 lowest, respectively, both in terms of their initial baseline Gini coefficients in 2011 and their average Gini coefficients across the 2011 and 2021 waves. Utilizing this dual criterion ensures the temporal stability of the income inequality contexts under investigation. The effective final analytical sample is 22 938 individuals.

### Measures

#### Dependent Variable: Individual Obesity

The dependent variable is a dichotomous measure of obesity status (1 = with obesity, 0 = without obesity) at the individual level, with an age-specific classification. For respondents aged 20 and over, obesity is defined as a Body Mass Index (BMI) of 30 or higher. For those aged 18 and 19, the classification follows the age- and sex-specific standards from the WHO Reference 2007, which sets a threshold slightly lower than 30. BMI was calculated from self-reported height and weight. To mitigate potential errors common in self-reported anthropometric data, we followed data-cleaning procedures adapted from established surveys such as the National Health and Nutrition Examination Survey (NHANES), the National Health Interview Survey (NHIS) and the Behavioral Risk Factor Surveillance System (BRFSS). Specifically, implausible self-reported values for height (eg, <50 inches or >90 inches) and weight were excluded. Weights below 80 lbs were removed, as were weights above 299 pounds for men and 274 pounds for women. These specific upper limits are adopted directly from the NHIS, which categorizes such values as representing “exceptionally high weight.” These upper thresholds for weight are conservative limits, as they are well above the 95th percentile of self-reported weight for each gender in major US health surveys.^15^ Given that the US has one of the highest obesity rates globally, applying these cut-offs to other countries is unlikely to unduly alter their sample compositions. Furthermore, individuals whose BMI fell outside of 3 standard deviations from their country’s mean were also removed from the sample. This step addresses potential measurement errors. Such errors often arise from respondents misinterpreting units (eg, kilograms vs pounds) or from other reporting inaccuracies that generate extreme outliers.

#### Independent Variable: Gini Coefficient

The primary independent variable is the Gini coefficient of disposable income, a country-level measure of income inequality ranging from 0 (perfect equality) to 100 (perfect inequality). Data are sourced from the Standardized World Income Inequality Database (SWIID), which provides comparable, high-quality data across countries and over time.

#### Control Variables

To isolate the effect of income inequality, our models include a range of control variables at both the individual and country levels. Individual-level controls include gender (categorized as female [reference] and male); age (continuous); educational attainment (categorized as primary or less [reference], secondary, and tertiary); marital status (categorized as married/civil partnership [reference], never married/single, separated or divorced, and widowed); employment status (categorized as in paid work [reference], unemployed and not in the labor force); household income (categorized in country-specific tertiles as lowest tertile [reference], middle tertile, highest tertile, and a category for missing income); the presence of a chronic illness or disability (categorized as no [reference] and yes); and household size (continuous). Country-level controls include GDP per capita, sourced from the IMF’s World Economic Outlook.

### Statistical Analysis

To test our hypothesis, we conduct regressions by subgroup, analyzing the high-Gini and low-Gini country groups separately. Given the panel structure of our data (individuals surveyed in 2 waves across multiple countries), it is crucial to control for unobserved country-specific factors that are stable over time (eg, cultural norms, healthcare system structure) and for general time trends affecting all countries. Failure to account for such factors could lead to omitted variable bias.

Therefore, we employ a Least Squares Dummy Variable Fixed Effects (LSDV-FE) model. This approach includes dummy variables for each country and for the survey year (2021 vs 2011), effectively controlling for all time-invariant, between-country heterogeneity and any secular trends common to the period. This allows us to isolate the effect of within-country changes in the Gini coefficient on the probability of obesity over the 10-year period.

We estimated the models separately for high-Gini and low-Gini country groups rather than using a pooled model with an interaction term, for 2 primary reasons. Econometrically, our strategy utilizes a two-way fixed-effects model that includes dummy variables for each country to control for time-invariant, between-country heterogeneity. Because a country’s classification into the high- or low-Gini group is a time-invariant characteristic based on its prevailing institutional context, a group dummy variable would be perfectly collinear with the country fixed effects. Consequently, the main effect of the inequality regime could not be estimated in a pooled fixed-effects framework. Theoretically, our framework posits that these country groups represent qualitatively distinct socio-political and welfare regimes. A pooled interaction model would unnecessarily constrain the coefficients of all control variables to be identical across these diverse societal contexts. Estimating the models separately by subgroup allows all covariate parameters to vary freely between the 2 regimes, offering a more nuanced and theoretically sound estimation of the relationships.

These variables are included in our models strictly as adjustment covariates (confounders) to account for compositional differences across countries and to minimize omitted variable bias. While individual socioeconomic factors, such as income and education, could plausibly function as effect modifiers in the relationship between national inequality and health, exploring such cross-level interactions falls outside the primary scope of this study. Our analytical focus remains on the macro-level modifying role of a country’s baseline inequality context.

For our primary analysis, we utilize a linear probability model (LPM). We selected the LPM primarily for the direct interpretability of its coefficients, which represent the change in the probability of obesity for a 1-unit change in the independent variable. To ensure our results are robust to this modeling choice, we also estimated fixed-effects logistic regression models, which yielded substantively identical conclusions.

## Results

This section presents the findings regarding the association between income inequality and obesity. It begins with descriptive statistics of the sample, followed by the results from the two-way fixed-effects models that test the study’s main hypothesis.

### Descriptive Statistics

Table 1 presents the descriptive statistics for the pooled analytical sample of 22 938 individuals from 10 countries across 2 survey waves (2011 and 2021). A detailed breakdown of the sample size for each country and wave is available in the Supplemental Materials (Table S1). Furthermore, detailed descriptive statistics stratified by each included country are provided in the Supplemental Materials (Tables S2a–S2j). The sample is relatively balanced in terms of gender and spans a diverse range of educational and employment backgrounds.

**Table 1.** Descriptive Statistics of the Analytical Sample (N = 22 938).

Variables	Prop./mean (SD)	Min.	Max.
Obesity			
No	82.9%	—	—
Yes	17.1%	—	—
Gender			
Female	52.3%	—	—
Male	47.7%	—	—
Age	47.62 (17.21)	18	100
Education			
Primary or less	21.2%	—	—
Secondary	44.3%	—	—
Tertiary	34.5%	—	—
Marital status			
Married, civil partnership	55.4%	—	—
Never married, single	27.5%	—	—
Separated or divorced	10.4%	—	—
Widowed	6.6%	—	—
Employment status			
In paid work	59.0%	—	—
Unemployed	4.4%	—	—
Not in labor force	36.6%	—	—
Income			
Lowest tertile	23.1%	—	—
Middle tertile	26.6%	—	—
Highest tertile	26.6%	—	—
Income missing	23.7%	—	—
Chronic illness or disability			
No	66.7%	—	—
Yes	33.3%	—	—
Household size	2.88 (1.53)	1	22
Observations	22 938		

*Source*. ISSP Health and Health Care I II, own calculations.

Table 2 outlines the country-level macroeconomic indicators, specifically mean GDP per capita and the Gini coefficients for both 2011 and 2021, alongside the decadal change in inequality for each nation. As detailed in Table 2, the low-Gini group displayed baseline coefficients ranging from 24.408 (Norway) to 25.434 (Slovenia) in 2011. Notably, this group experienced substantive within-country variation over the decade, such as a 3.372-point increase in Norway and a 2.694-point decrease in Slovakia. The high-Gini group exhibited baseline 2011 coefficients between 32.764 (Australia) and 38.037 (United States), with varying degrees of subsequent fluctuation, including a 4.994-point decrease in Russia and a 2.788-point decrease in Israel. This descriptive evidence provides the empirical foundation for the subsequent fixed-effects models, which exploit precisely this within-country variation to estimate the conditional effect of rising inequality on obesity across the 2 inequality regimes.

**Table 2.** Country-Level Macroeconomic Indicators.

Country	Mean GDP per capita	Gini 2011	Gini 2021	Gini (2021-2011)
Australia	65.77037	32.764	32.626	−0.137
Czechia	24.86822	25.084	24.778	−0.307
Finland	52.18929	25.362	26.367	1.005
Israel	43.41413	36.980	34.192	−2.788
Italy	37.64436	33.001	33.219	0.219
Norway	96.88559	24.408	27.780	3.372
Russia	13.36674	35.989	30.995	−4.994
Slovakia	20.29428	25.026	22.332	−2.694
Slovenia	27.08141	25.434	24.136	−1.297
United States	60.61983	38.037	37.556	−0.480

### Multivariate Analysis

Table 3 presents the results of the two-way fixed-effects linear probability models. Following our analytical strategy, the countries were categorized into high-Gini and low-Gini groups to test for a conditional relationship.

**Table 3.** Results of Two-Way Fixed-Effects Linear Probability Models Predicting Obesity Status.

Variables	Model 1	Model 2
Gini coefficient	0.020** [0.007, 0.032]	−0.005 [−0.012, 0.002]
Gender		
Female	Ref.	Ref.
Male	0.016* [0.003, 0.030]	−0.018* [−0.033, −0.004]
Age	0.002*** [0.001, 0.002]	0.002*** [0.001, 0.002]
Education		
Primary or less	Ref.	Ref.
Secondary	−0.019* [−0.037, −0.001]	−0.018 [−0.039, 0.004]
Tertiary	−0.056*** [−0.076, −0.036]	−0.063*** [−0.086, −0.039]
Marital status		
Married, civil partnership	Ref.	Ref.
Never married, single	−0.015 [−0.034, 0.004]	−0.030** [−0.050, −0.009]
Separated or divorced	−0.006 [−0.030, 0.017]	−0.019 [−0.042, 0.005]
Widowed	0.023 [−0.008, 0.053]	−0.004 [−0.034, 0.027]
Employment status		
In paid work	Ref.	Ref.
Unemployed	0.023 [−0.014, 0.060]	−0.001 [−0.035, 0.033]
Not in labor force	−0.013 [−0.029, 0.003]	−0.022* [−0.039, −0.004]
Income		
Lowest tertile	Ref.	Ref.
Middle tertile	−0.013 [−0.033, 0.007]	−0.021* [−0.042, −0.001]
Highest tertile	−0.025* [−0.047, −0.003]	−0.033** [−0.055, −0.011]
Income missing	−0.019 [−0.039, 0.001]	−0.052*** [−0.075, −0.030]
Household size	0.007* [0.001, 0.012]	0.008** [0.003, 0.014]
Chronic illness or disability		
No	Ref.	Ref.
Yes	0.098*** [0.084, 0.113]	0.087*** [0.071, 0.103]
GDP per capita	0.006* [0.001, 0.011]	−0.000 [−0.001, 0.001]
Constant	−0.606** [−1.013, −0.200]	0.339** [0.090, 0.589]
Two-way FE	Yes	Yes
Number of countries	5	5
Number of country-years	10	10
Observations	11 586	11 352
Adjusted R-squared	0.042	0.050

*Note*. 95% confidence intervals in brackets.

*** *P* < .001. **P < .01. *P < .05.

Consistent with our hypothesis, the findings reveal that the association between income inequality and obesity is conditional on the country’s baseline level of income inequality. Model 1, which analyzes the low-Gini country group, shows a positive and statistically significant coefficient for the Gini coefficient (β = .020, *P* < .01). This indicates that in these more egalitarian societies, a 1-unit within-country increase in the Gini coefficient is associated with a 2.0 percentage point increase in the probability of being obese, holding all other factors constant.

Conversely, and also in line with our hypothesis, Model 2 shows no statistically significant association between the Gini coefficient and obesity in the high-Gini country group (β = −.005, *P* > .10). In societies where a high level of inequality is already entrenched, further increases in the Gini coefficient do not produce a distinguishable effect on the likelihood of obesity.

### Robustness Checks

To ensure the robustness of our findings, we conducted several additional analyses, which are detailed in the Supplemental Materials. We first re-estimated our models using logistic regression, and the results, presented as odds ratios in Models 3 and 4 (Table S3), were consistent with our primary findings from the linear probability models. The positive and significant association between the Gini coefficient and obesity held in the low-inequality countries (OR = 1.171, *P* < .01), while the association remained non-significant in the high-inequality countries. Further models tested for the influence of potential outlier countries. Model 5, which excluded Slovakia (the country with the lowest Gini coefficient) from the low-Gini group, and Model 6 (Table S4), which excluded the United States (the country with the highest Gini coefficient) from the high-Gini group, produced substantively identical results. Additionally, models that expanded the country samples by adding the Netherlands to the low-Gini group (Model 7) and France to the high-Gini group (Model 8) also confirmed the stability of our initial results (Table S5). These additional tests support to our conclusion that the relationship between income inequality and obesity is contingent on a country’s existing level of inequality. Finally, given the relatively large proportion of respondents missing data for household income (23.7%), we conducted a sensitivity analysis to ensure our findings were not an artifact of our missing data handling strategy. We employed multiple imputation by chained equations (MICE), generating 5 imputed datasets to account for missing income values. The results from these imputed models, presented in Table S6 (Models 9 and 10), are substantively identical to our primary findings. The association between the Gini coefficient and obesity remains positive and highly significant in the low-Gini group and non-significant in the high-Gini group, further confirming the stability of the conditional effect.

**Figure 1.**
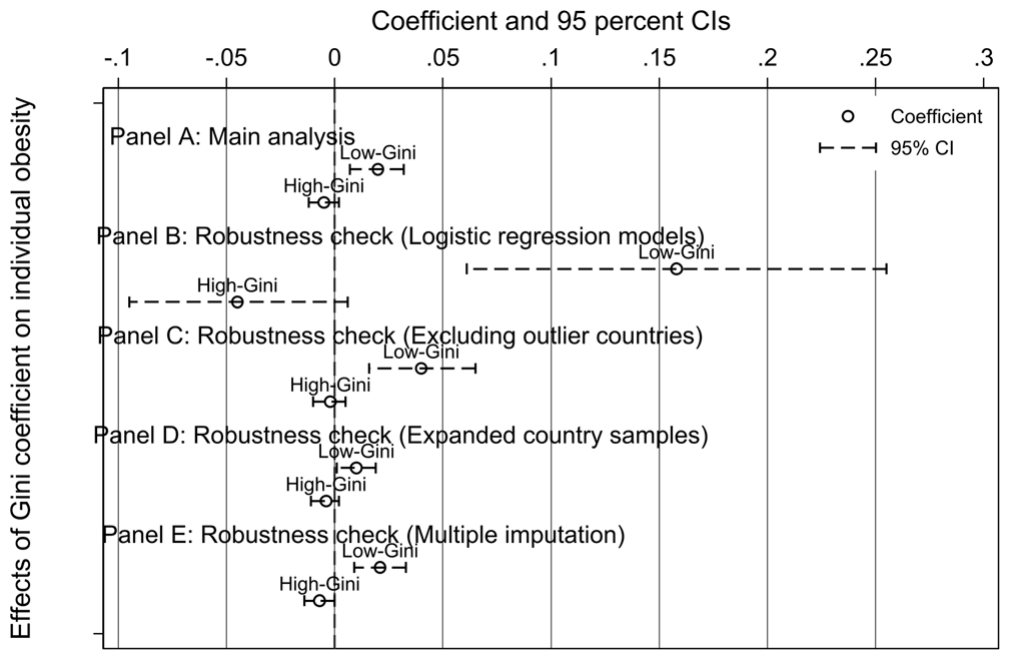
Forest plot of the estimated effect of the Gini coefficient on obesity across all model specifications.

To provide a comprehensive visual summary and demonstrate the stability of our main conclusion, Figure 1 presents a forest plot of the estimated coefficients for the Gini coefficient from all 10 models. The plot visually confirms the central finding: the coefficient for the Gini coefficient is consistently positive and statistically significant in the low-inequality contexts (Models 1, 3, 5, 7 and 9), an effect that holds across alternative estimation methods, and sample specifications and missing data handling strategies. In contrast, the effect remains statistically indistinguishable from zero across all corresponding models for the high-inequality group (Models 2, 4, 6, 8 and 10). This visualization underscores the robustness of our central finding that the relationship between rising income inequality and obesity is conditional, emerging as a significant health risk only in societies with a more egalitarian baseline.

## Discussion

This study investigated the conditional relationship between income inequality and obesity across 2 distinct groups of affluent nations. The findings provide robust support for our central hypothesis: the impact of rising income inequality on residents’ probability of obesity is not uniform but is contingent on a nation’s baseline level of income inequality. In countries with historically low Gini coefficients, an increase in inequality was significantly associated with a higher probability of obesity. Conversely, in countries where high inequality is already entrenched, a similar increase in the Gini coefficient had no statistically distinguishable effect. This finding helps reconcile the mixed results in the existing literature^11-13^ and advances understanding of the pathways linking societal context to population health.

The positive association in the low-Gini countries lends credence to theories emphasizing the disruptive power of growing social distances. From a psychosocial perspective, a shift toward greater income disparity in a relatively egalitarian society may function as a salient social shock. In such contexts, where social comparisons are typically made within a more compressed social hierarchy,^16^ the experience of relative deprivation can become more acute, intensifying status anxiety and competitive consumption.^6^ This chronic stress, which has been robustly linked to subordinate social status,^17^ can have direct physiological consequences, such as elevated cortisol levels, which are known to promote abdominal fat storage and stimulate appetite for energy-dense “comfort” foods.^18^ A neo-materialist pathway complements this psychosocial mechanism. Rising inequality may signal a political shift away from collective investment in public goods and social infrastructure, such as public recreation facilities and healthy food subsidies. This shift can lead to the gradual emergence of a more obesogenic environment, where unhealthy choices become the default for many.^9,19^

The null finding in the high-Gini countries is equally informative, pointing beyond a simple saturation effect to a process we term **ideological buffering**. A saturation effect would imply a deterministic mathematical ceiling, suggesting that income inequality has simply reached a maximal limit beyond which further increases cannot inflict additional observable harm. While existing literature acknowledges that the health impacts of inequality can be non-linear, it may be premature and overly reductionist to attribute the lack of association in high-Gini nations solely to such a statistical ceiling. Instead, we propose that this attenuation is inextricably linked to the broader socio-cultural context and the chronic nature of the inequality itself. In these societies, high inequality is not merely an economic condition but is often legitimized by dominant national narratives of meritocracy and individualism.^20,21^ Such ideologies can function as a psychosocial buffer, encouraging citizens to interpret social stratification as a fair outcome of individual talent and effort, rather than a systemic failure. This aligns with system justification theory, which posits that individuals are motivated to defend and rationalize the status quo, even when it is disadvantageous to them.^22^ This process of legitimation may blunt the collective experience of status anxiety and social friction, leading individuals to internalize blame for their socioeconomic position rather than directing frustration at a systemic problem. In contrast, in the low-Gini countries, often characterized by social democratic traditions and stronger norms of solidarity,^23^ a rise in inequality is more likely to be perceived as a violation of the social contract. This perception of systemic injustice generates the very insecurity and erosion of trust that activate health-damaging pathways. Thus, the crucial difference lies not only in the economic structure but in the ideological framework through which that structure is understood and experienced.

This study has several strengths, including the use of high-quality, individual-level data from 2 waves of the ISSP, which allows for the control of individual-level confounders and mitigates the risk of ecological fallacy. Furthermore, our use of a two-way fixed-effects model isolates the effect of within-country changes over time, providing a more rigorous test of the hypothesis than cross-sectional designs. Nevertheless, the study is not without limitations. First, the measure of obesity is based on self-reported height and weight, which may be subject to social desirability bias and lead to underestimation of true BMI.^24^ Furthermore, the magnitude of this reporting bias may vary across different cultural contexts due to distinct norms regarding body size, which could potentially influence cross-national comparisons. Second, the Gini coefficient, while a standard measure, is a summary statistic that is most sensitive to changes in the middle of the income distribution and may not fully capture the impact of wealth concentration at the very top, which some scholars argue is a more potent driver of social and political dysfunction.^25^ Future research could explore how different facets of inequality, such as the income share of the top 1%, relate to health outcomes. Finally, while our comparative approach is a key strength, the 10-year period between survey waves may not be long enough to capture the full, lagged effects of socioeconomic changes on a chronic condition like obesity. With only 2 time points, we cannot assess whether inequality–obesity associations emerge with longer lags, nor can we fully characterize trajectories of change. In addition, fixed-effects estimation with a small number of country-years may reduce statistical power and yield wider confidence intervals, particularly in contexts where within-country variation in inequality is limited. Future research with additional waves would be better positioned to examine longer-run dynamics and to improve precision. Beyond incorporating more time points, subsequent investigations should also explore potential cross-level interactions to determine whether individual-level socioeconomic factors, such as education or income, act as effect modifiers in the relationship between national inequality and obesity.

Despite these limitations, the findings have important and distinct implications. For future research, our results highlight the need to move beyond a search for a universal effect of inequality and instead adopt context-sensitive approaches that investigate the conditions under which it matters most. Multi-level studies incorporating community-level data (eg, food environments, access to green space) could help to further disentangle the neo-materialist and psychosocial pathways.

For policy, our findings suggest that a uniform approach is inadequate. Low-inequality nations already benefit from universal public infrastructures and comprehensive social services. In these contexts, primary prevention should prioritize macro-level policies that prevent income gaps from widening, such as maintaining progressive tax structures and strong labor protections, to preserve these established health-promoting environments. Conversely, in high-inequality nations where access to such broad social provisions is often highly stratified or unevenly distributed, minor fluctuations in the Gini coefficient are unlikely to yield measurable health benefits. In these settings, deep structural interventions, including expanding equitable public education, universal healthcare access, and robust social safety nets, are necessary to address the entrenched institutional consequences of systemic inequality.

## Conclusion

In conclusion, this study demonstrates that the relationship between income inequality and obesity is context-dependent. The health consequences of rising inequality are most pronounced in societies where equality has historically been the norm. This underscores the critical importance of considering how both the baseline level and the change in income inequality interact to shape population health. Ultimately, inequality casts its longest shadow on health not when it is a familiar feature of the landscape, but when it begins to disrupt a previously egalitarian environment.

## Supplemental Material

sj-docx-1-inq-10.1177_00469580261444418 – Supplemental material for The Conditional Effect of Income Inequality on Obesity: A Cross-National Comparative Analysis Supplemental material, sj-docx-1-inq-10.1177_00469580261444418 for The Conditional Effect of Income Inequality on Obesity: A Cross-National Comparative Analysis by Qian Hu, Wei Zhong and Rong Hu in INQUIRY: The Journal of Health Care Organization, Provision, and Financing

sj-docx-2-inq-10.1177_00469580261444418 – Supplemental material for The Conditional Effect of Income Inequality on Obesity: A Cross-National Comparative Analysis Supplemental material, sj-docx-2-inq-10.1177_00469580261444418 for The Conditional Effect of Income Inequality on Obesity: A Cross-National Comparative Analysis by Qian Hu, Wei Zhong and Rong Hu in INQUIRY: The Journal of Health Care Organization, Provision, and Financing

## References

[1] World Health Organization. Obesity and overweight. Published December 8, 2025. Accessed January 10, 2026. https://www.who.int/news-room/fact-sheets/detail/obesity-and-overweight

[2] Link BG Phelan J. Social conditions as fundamental causes of disease. J Health Soc Behav. 1995;35:80-94. doi:10.2307/2626958 7560851

[3] Marmot M. Social determinants of health inequalities. Lancet. 2005;365(9464):1099-1104. doi:10.1016/S0140-6736(05)71146-6 15781105

[4] Sobal J Stunkard AJ. Socioeconomic status and obesity: a review of the literature. Psychol Bull. 1989;105(2):260-275. doi:10.1037/0033-2909.105.2.260 2648443

[5] McLaren L. Socioeconomic status and obesity. Epidemiol Rev. 2007;29(1):29-48. doi:10.1093/epirev/mxm001 17478442

[6] Wilkinson R Pickett K. The Spirit Level. Penguin Books; 2009.

[7] Layte R Whelan CT. Who feels inferior? A test of the status anxiety hypothesis of social inequalities in health. Eur Sociol Rev. 2014;30(4):525-535. doi:10.1093/esr/jcu057

[8] Pickett KE Wilkinson RG. Income inequality and health: a causal review. Soc Sci Med. 2015;128:316-326. doi:10.1016/j.socscimed.2014.12.031 25577953

[9] Lynch JW Smith GD Kaplan GA House JS. Income inequality and mortality: importance to health of individual income, psychosocial environment, or material conditions. BMJ. 2000; 320(7243):1200-1204. doi:10.1136/bmj.320.7243.1200 10784551 PMC1127589

[10] Offer A Pechey R Ulijaszek S. Insecurity, Inequality, and Obesity in Affluent Societies. British Academy; 2012.

[11] Pickett KE Kelly S Brunner E Lobstein T Wilkinson RG. Wider income gaps, wider waistbands? An ecological study of obesity and income inequality. J Epidemiol Community Health. 2005;59(8):670-674. doi:10.1136/jech.2004.028795 16020644 PMC1733121

[bibr12-00469580261444418] Su D Esqueda OA Li L Pagán JA. Income inequality and obesity prevalence among OECD countries. J Biosoc Sci. 2012;44(4):417-432. doi:10.1017/S002193201100071X 22214551

[13] Clément M Levasseur P Seetahul S Piaser L. Does inequality have a silver lining? Municipal income inequality and obesity in Mexico. Soc Sci Med. 2021;272:113710. doi:10.1016/j.socscimed.2021.113710 33516086

[14] ISSP Research Group. Data from: International Social Survey Programme: Health and Health Care I-II Cumulation. 2024. doi:10.4232/1.14438

[15] Flegal KM Ogden CL Fryar C Afful J Klein R Huang DT . Comparisons of self-reported and measured height and weight, BMI, and obesity prevalence from National Surveys: 1999-2016. Obesity. 2019/10/01;27(10):1711-1719. doi:10.1002/oby.22591 PMC7289317 31544344

[16] Frank RH. Falling Behind: How Rising Inequality Harms the Middle Class. University of California Press; 2007.

[17] Sapolsky RM . The influence of social hierarchy on Primate Health. Science. 2005/04/29;308(5722):648-652. doi:10.1126/science.1106477 15860617

[18] Brunner E Marmot M . Social organization, stress, and health. In: Marmot M, Wilkinson RG, eds. Social Determinants of Health. 2nd ed. Oxford University Press; 2006;6-30.

[19] Papas MA Alberg AJ Ewing R Helzlsouer KJ Gary TL Klassen AC. The built environment and obesity. Epidemiol Rev. 2007;29(1):129-143. doi:10.1093/epirev/mxm009 17533172

[20] Lamont M. From ‘having’ to ‘being’: self-worth and the current crisis of American society. Br J Sociol. 2019;70(3):660-707. doi:10.1111/1468-4446.12667 31190392

[21] Mijs JJB . The paradox of inequality: income inequality and belief in meritocracy go hand in hand. Socioecon Rev. 2021; 19(1):7-35. doi:10.1093/ser/mwy051

[22] Jost JT Banaji MR. The role of stereotyping in system-justification and the production of false consciousness. Br J Soc Psychol. 1994;33(1):1-27. doi:10.1111/j.2044-8309.1994.tb01008.x

[23] Rothstein B Uslaner EM. All for all: equality, corruption, and social trust. World Polit. 2005;58(1):41-72. doi:10.1353/wp.2006.0022

[24] Gorber SC Tremblay M Moher D Gorber B. A comparison of direct vs. self-report measures for assessing height, weight and body mass index: a systematic review. Obes Rev. 2007; 8(4):307-326. doi:10.1111/j.1467-789X.2007.00347.x 17578381

[25] Atkinson AB Piketty T Saez E. Top incomes in the long run of History. J Econ Lit. 2011;49(1):3-71. doi:10.1257/jel.49.1.3

