# Supplementary material for: The Conditional Effect of Income Inequality on Obesity: A Cross-National Comparative Analysis
Source: Inquiry. 2026 Apr 21;63:00469580261444418. doi: 10.1177/00469580261444418 (PMC13111845; doi:10.1177/00469580261444418)
Supplement: sj-docx-2-inq-10.1177_00469580261444418 – Supplemental material for The Conditional Effect of Income Inequality on Obesity: A Cross-National Comparative Analysis [file sj-docx-2-inq-10.1177_00469580261444418.docx]

Table S1. Sample Distribution by Country and Survey Year

|  | Years for each ISSP round | | |
| --- | --- | --- | --- |
|  | 2011 | 2021 | Total |
| Australia | 1,351 | 741 | 2,092 |
| Czechia | 1,512 | 1,169 | 2,681 |
| Finland | 1,162 | 815 | 1,977 |
| Israel | 1,097 | 1,070 | 2,167 |
| Italy | 1,016 | 875 | 1,891 |
| Norway | 1,663 | 1,310 | 2,973 |
| Russia | 1,332 | 1,493 | 2,825 |
| Slovakia | 1,089 | 858 | 1,947 |
| Slovenia | 1,035 | 973 | 2,008 |
| United States | 1,408 | 969 | 2,377 |
| Total | 12,665 | 10,273 | 22,938 |

Table S2a. Descriptive Statistics for Australia

|  | Prop./Mean (SD) | Min. | Max. |
| --- | --- | --- | --- |
| Obesity |  |  |  |
| No | 76.6% | — | — |
| Yes | 23.4% | — | — |
| Gender |  |  |  |
| Female | 48.2% | — | — |
| Male | 51.8% | — | — |
| Age | 46.24 (17.25) | 18 | 93 |
| Education |  |  |  |
| Primary or less | 23.3% | — | — |
| Secondary | 37.9% | — | — |
| Tertiary | 38.9% | — | — |
| Marital status |  |  |  |
| Married, civil partnership | 56.5% | — | — |
| Never married, single | 31.9% | — | — |
| Separated or divorced | 8.1% | — | — |
| Widowed | 3.5% | — | — |
| Employment status |  |  |  |
| In paid work | 63.4% | — | — |
| Unemployed | 3.3% | — | — |
| Not in labor force | 33.4% | — | — |
| Income |  |  |  |
| Lowest tertile | 20.4% | — | — |
| Middle tertile | 25.7% | — | — |
| Highest tertile | 28.5% | — | — |
| Income missing | 25.4% | — | — |
| Chronic illness or disability |  |  |  |
| No | 68.6% | — | — |
| Yes | 31.4% | — | — |
| Household size | 3.05 (1.51) | 1 | 12 |
| Observations | 2,092 |  |  |
| Source: ISSP Health and Health Care I II, own calculations. | | | |

Table S2b. Descriptive Statistics for Czechia

|  | Prop./Mean (SD) | Min. | Max. |
| --- | --- | --- | --- |
| Obesity |  |  |  |
| No | 85.8% | — | — |
| Yes | 14.2% | — | — |
| Gender |  |  |  |
| Female | 51.6% | — | — |
| Male | 48.4% | — | — |
| Age | 47.73 (17.60) | 18 | 92 |
| Education |  |  |  |
| Primary or less | 29.0% | — | — |
| Secondary | 54.1% | — | — |
| Tertiary | 16.9% | — | — |
| Marital status |  |  |  |
| Married, civil partnership | 51.2% | — | — |
| Never married, single | 25.8% | — | — |
| Separated or divorced | 13.8% | — | — |
| Widowed | 9.1% | — | — |
| Employment status |  |  |  |
| In paid work | 58.4% | — | — |
| Unemployed | 3.9% | — | — |
| Not in labor force | 37.7% | — | — |
| Income |  |  |  |
| Lowest tertile | 19.2% | — | — |
| Middle tertile | 22.4% | — | — |
| Highest tertile | 26.7% | — | — |
| Income missing | 31.7% | — | — |
| Chronic illness or disability |  |  |  |
| No | 73.2% | — | — |
| Yes | 26.8% | — | — |
| Household size | 2.56 (1.25) | 1 | 10 |
| Observations | 2,681 |  |  |
| Source: ISSP Health and Health Care I II, own calculations. | | | |

Table S2c. Descriptive Statistics for Finland

|  | Prop./Mean (SD) | Min. | Max. |
| --- | --- | --- | --- |
| Obesity |  |  |  |
| No | 85.3% | — | — |
| Yes | 14.7% | — | — |
| Gender |  |  |  |
| Female | 50.6% | — | — |
| Male | 49.4% | — | — |
| Age | 46.52 (16.35) | 18 | 75 |
| Education |  |  |  |
| Primary or less | 13.5% | — | — |
| Secondary | 46.5% | — | — |
| Tertiary | 40.1% | — | — |
| Marital status |  |  |  |
| Married, civil partnership | 50.4% | — | — |
| Never married, single | 40.1% | — | — |
| Separated or divorced | 7.4% | — | — |
| Widowed | 2.1% | — | — |
| Employment status |  |  |  |
| In paid work | 59.6% | — | — |
| Unemployed | 4.0% | — | — |
| Not in labor force | 36.5% | — | — |
| Income |  |  |  |
| Lowest tertile | 21.8% | — | — |
| Middle tertile | 27.7% | — | — |
| Highest tertile | 28.8% | — | — |
| Income missing | 21.7% | — | — |
| Chronic illness or disability |  |  |  |
| No | 55.7% | — | — |
| Yes | 44.3% | — | — |
| Household size | 2.48 (1.27) | 1 | 10 |
| Observations | 1,977 |  |  |
| Source: ISSP Health and Health Care I II, own calculations. | | | |

Table S2d. Descriptive Statistics for Israel

|  | Prop./Mean (SD) | Min. | Max. |
| --- | --- | --- | --- |
| Obesity |  |  |  |
| No | 83.1% | — | — |
| Yes | 16.9% | — | — |
| Gender |  |  |  |
| Female | 53.5% | — | — |
| Male | 46.5% | — | — |
| Age | 45.63 (16.84) | 18 | 94 |
| Education |  |  |  |
| Primary or less | 20.1% | — | — |
| Secondary | 38.1% | — | — |
| Tertiary | 41.8% | — | — |
| Marital status |  |  |  |
| Married, civil partnership | 64.0% | — | — |
| Never married, single | 23.8% | — | — |
| Separated or divorced | 8.0% | — | — |
| Widowed | 4.1% | — | — |
| Employment status |  |  |  |
| In paid work | 66.9% | — | — |
| Unemployed | 3.9% | — | — |
| Not in labor force | 29.2% | — | — |
| Income |  |  |  |
| Lowest tertile | 24.7% | — | — |
| Middle tertile | 25.4% | — | — |
| Highest tertile | 25.2% | — | — |
| Income missing | 24.7% | — | — |
| Chronic illness or disability |  |  |  |
| No | 67.6% | — | — |
| Yes | 32.4% | — | — |
| Household size | 3.75 (1.96) | 1 | 17 |
| Observations | 2,167 |  |  |
| Source: ISSP Health and Health Care I II, own calculations. | | | |

Table S2e. Descriptive Statistics for Italy

|  | Prop./Mean (SD) | Min. | Max. |
| --- | --- | --- | --- |
| Obesity |  |  |  |
| No | 90.0% | — | — |
| Yes | 10.0% | — | — |
| Gender |  |  |  |
| Female | 50.5% | — | — |
| Male | 49.5% | — | — |
| Age | 52.88 (18.12) | 18 | 94 |
| Education |  |  |  |
| Primary or less | 42.8% | — | — |
| Secondary | 39.4% | — | — |
| Tertiary | 17.7% | — | — |
| Marital status |  |  |  |
| Married, civil partnership | 58.1% | — | — |
| Never married, single | 26.3% | — | — |
| Separated or divorced | 6.4% | — | — |
| Widowed | 9.2% | — | — |
| Employment status |  |  |  |
| In paid work | 47.4% | — | — |
| Unemployed | 7.9% | — | — |
| Not in labor force | 44.7% | — | — |
| Income |  |  |  |
| Lowest tertile | 28.9% | — | — |
| Middle tertile | 26.1% | — | — |
| Highest tertile | 21.7% | — | — |
| Income missing | 23.4% | — | — |
| Chronic illness or disability |  |  |  |
| No | 65.3% | — | — |
| Yes | 34.7% | — | — |
| Household size | 2.65 (1.22) | 1 | 9 |
| Observations | 1,891 |  |  |
| Source: ISSP Health and Health Care I II, own calculations. | | | |

Table S2f. Descriptive Statistics for Norway

|  | Prop./Mean (SD) | Min. | Max. |
| --- | --- | --- | --- |
| Obesity |  |  |  |
| No | 84.8% | — | — |
| Yes | 15.2% | — | — |
| Gender |  |  |  |
| Female | 53.5% | — | — |
| Male | 46.5% | — | — |
| Age | 48.61 (16.06) | 18 | 79 |
| Education |  |  |  |
| Primary or less | 17.9% | — | — |
| Secondary | 29.4% | — | — |
| Tertiary | 52.7% | — | — |
| Marital status |  |  |  |
| Married, civil partnership | 56.1% | — | — |
| Never married, single | 30.3% | — | — |
| Separated or divorced | 10.3% | — | — |
| Widowed | 3.2% | — | — |
| Employment status |  |  |  |
| In paid work | 65.6% | — | — |
| Unemployed | 1.3% | — | — |
| Not in labor force | 33.1% | — | — |
| Income |  |  |  |
| Lowest tertile | 29.4% | — | — |
| Middle tertile | 28.4% | — | — |
| Highest tertile | 31.3% | — | — |
| Income missing | 10.9% | — | — |
| Chronic illness or disability |  |  |  |
| No | 62.4% | — | — |
| Yes | 37.6% | — | — |
| Household size | 2.63 (1.59) | 1 | 22 |
| Observations | 2,973 |  |  |
| Source: ISSP Health and Health Care I II, own calculations. | | | |

Table S2g. Descriptive Statistics for Russia

|  | Prop./Mean (SD) | Min. | Max. |
| --- | --- | --- | --- |
| Obesity |  |  |  |
| No | 82.1% | — | — |
| Yes | 17.9% | — | — |
| Gender |  |  |  |
| Female | 57.6% | — | — |
| Male | 42.4% | — | — |
| Age | 46.04 (17.06) | 18 | 91 |
| Education |  |  |  |
| Primary or less | 6.3% | — | — |
| Secondary | 44.2% | — | — |
| Tertiary | 49.5% | — | — |
| Marital status |  |  |  |
| Married, civil partnership | 45.0% | — | — |
| Never married, single | 26.0% | — | — |
| Separated or divorced | 15.6% | — | — |
| Widowed | 13.3% | — | — |
| Employment status |  |  |  |
| In paid work | 57.2% | — | — |
| Unemployed | 5.1% | — | — |
| Not in labor force | 37.7% | — | — |
| Income |  |  |  |
| Lowest tertile | 25.4% | — | — |
| Middle tertile | 29.7% | — | — |
| Highest tertile | 27.2% | — | — |
| Income missing | 17.7% | — | — |
| Chronic illness or disability |  |  |  |
| No | 67.1% | — | — |
| Yes | 32.9% | — | — |
| Household size | 2.47 (1.28) | 1 | 10 |
| Observations | 2,825 |  |  |
| Source: ISSP Health and Health Care I II, own calculations. | | | |

Table S2h. Descriptive Statistics for Slovakia

|  | Prop./Mean (SD) | Min. | Max. |
| --- | --- | --- | --- |
| Obesity |  |  |  |
| No | 84.5% | — | — |
| Yes | 15.5% | — | — |
| Gender |  |  |  |
| Female | 52.0% | — | — |
| Male | 48.0% | — | — |
| Age | 45.84 (16.89) | 18 | 92 |
| Education |  |  |  |
| Primary or less | 28.7% | — | — |
| Secondary | 53.1% | — | — |
| Tertiary | 18.1% | — | — |
| Marital status |  |  |  |
| Married, civil partnership | 56.7% | — | — |
| Never married, single | 28.7% | — | — |
| Separated or divorced | 7.8% | — | — |
| Widowed | 6.7% | — | — |
| Employment status |  |  |  |
| In paid work | 58.4% | — | — |
| Unemployed | 6.1% | — | — |
| Not in labor force | 35.5% | — | — |
| Income |  |  |  |
| Lowest tertile | 13.1% | — | — |
| Middle tertile | 24.2% | — | — |
| Highest tertile | 21.1% | — | — |
| Income missing | 41.6% | — | — |
| Chronic illness or disability |  |  |  |
| No | 72.2% | — | — |
| Yes | 27.8% | — | — |
| Household size | 3.34 (1.52) | 1 | 12 |
| Observations | 1,947 |  |  |
| Source: ISSP Health and Health Care I II, own calculations. | | | |

Table S2i. Descriptive Statistics for Slovenia

|  | Prop./Mean (SD) | Min. | Max. |
| --- | --- | --- | --- |
| Obesity |  |  |  |
| No | 83.4% | — | — |
| Yes | 16.6% | — | — |
| Gender |  |  |  |
| Female | 51.0% | — | — |
| Male | 49.0% | — | — |
| Age | 48.98 (18.08) | 18 | 100 |
| Education |  |  |  |
| Primary or less | 23.9% | — | — |
| Secondary | 49.2% | — | — |
| Tertiary | 27.0% | — | — |
| Marital status |  |  |  |
| Married, civil partnership | 69.6% | — | — |
| Never married, single | 18.7% | — | — |
| Separated or divorced | 4.7% | — | — |
| Widowed | 7.0% | — | — |
| Employment status |  |  |  |
| In paid work | 50.0% | — | — |
| Unemployed | 4.2% | — | — |
| Not in labor force | 45.8% | — | — |
| Income |  |  |  |
| Lowest tertile | 18.1% | — | — |
| Middle tertile | 20.3% | — | — |
| Highest tertile | 19.4% | — | — |
| Income missing | 42.3% | — | — |
| Chronic illness or disability |  |  |  |
| No | 69.0% | — | — |
| Yes | 31.0% | — | — |
| Household size | 3.21 (1.46) | 1 | 12 |
| Observations | 2,008 |  |  |
| Source: ISSP Health and Health Care I II, own calculations. | | | |

Table S2j. Descriptive Statistics for United States

|  | Prop./Mean (SD) | Min. | Max. |
| --- | --- | --- | --- |
| Obesity |  |  |  |
| No | 74.0% | — | — |
| Yes | 26.0% | — | — |
| Gender |  |  |  |
| Female | 52.3% | — | — |
| Male | 47.7% | — | — |
| Age | 48.27 (17.06) | 18 | 89 |
| Education |  |  |  |
| Primary or less | 14.1% | — | — |
| Secondary | 53.9% | — | — |
| Tertiary | 32.0% | — | — |
| Marital status |  |  |  |
| Married, civil partnership | 51.5% | — | — |
| Never married, single | 24.4% | — | — |
| Separated or divorced | 17.4% | — | — |
| Widowed | 6.7% | — | — |
| Employment status |  |  |  |
| In paid work | 59.0% | — | — |
| Unemployed | 5.7% | — | — |
| Not in labor force | 35.3% | — | — |
| Income |  |  |  |
| Lowest tertile | 26.5% | — | — |
| Middle tertile | 33.6% | — | — |
| Highest tertile | 32.7% | — | — |
| Income missing | 7.2% | — | — |
| Chronic illness or disability |  |  |  |
| No | 65.3% | — | — |
| Yes | 34.7% | — | — |
| Household size | 2.93 (1.59) | 1 | 11 |
| Observations | 2,377 |  |  |
| Source: ISSP Health and Health Care I II, own calculations. | | | |

Table S3. Results of Two-Way Fixed-Effects Logistic Regression Models Predicting Obesity Status

|  | Model 3 | Model 4 |
| --- | --- | --- |
| Gini coefficient | 1.171** | 0.956 |
|  | [1.063, 1.290] | [0.909, 1.006] |
| Gender |  |  |
| Female | Ref. | Ref. |
| Male | 1.138* | 0.877* |
|  | [1.023, 1.265] | [0.793, 0.969] |
| Age | 1.016*** | 1.012*** |
|  | [1.011, 1.021] | [1.008, 1.017] |
| Education |  |  |
| Primary or less | Ref. | Ref. |
| Secondary | 0.869* | 0.872 |
|  | [0.757, 0.997] | [0.756, 1.005] |
| Tertiary | 0.635*** | 0.644*** |
|  | [0.538, 0.748] | [0.553, 0.751] |
| Marital status |  |  |
| Married, civil partnership | Ref. | Ref. |
| Never married, single | 0.843* | 0.772*** |
|  | [0.716, 0.994] | [0.665, 0.896] |
| Separated or divorced | 0.960 | 0.868 |
|  | [0.799, 1.153] | [0.743, 1.013] |
| Widowed | 1.125 | 0.934 |
|  | [0.911, 1.390] | [0.773, 1.128] |
| Employment status |  |  |
| In paid work | Ref. | Ref. |
| Unemployed | 1.207 | 0.996 |
|  | [0.910, 1.600] | [0.782, 1.267] |
| Not in labor force | 0.859* | 0.836** |
|  | [0.753, 0.979] | [0.741, 0.944] |
| Income |  |  |
| Lowest tertile | Ref. | Ref. |
| Middle tertile | 0.911 | 0.878 |
|  | [0.780, 1.064] | [0.769, 1.001] |
| Highest tertile | 0.826* | 0.810** |
|  | [0.693, 0.984] | [0.699, 0.940] |
| Income missing | 0.867 | 0.687*** |
|  | [0.744, 1.011] | [0.586, 0.805] |
| Household size | 1.053* | 1.059** |
|  | [1.011, 1.097] | [1.021, 1.098] |
| Chronic illness or disability |  |  |
| No | Ref. | Ref. |
| Yes | 2.045*** | 1.740*** |
|  | [1.830, 2.285] | [1.569, 1.929] |
| GDP per capita | 1.052* | 1.000 |
|  | [1.012, 1.093] | [0.991, 1.009] |
| Constant | 0.000*** | 0.820 |
|  | [0.000, 0.009] | [0.151, 4.463] |
| Two-way FE | Yes | Yes |
| Number of countries | 5 | 5 |
| Number of country-years | 10 | 10 |
| Observations | 11,586 | 11,352 |
| Pseudo R-squared | 0.051 | 0.055 |
| *** p<.001, ** p<.01, * p<.05. 95% confidence intervals in brackets. | | |

Table S4. Results of Two-Way Fixed-Effects Linear Probability Models Predicting Obesity Status, Excluding Outlier Countries

|  | Model 5 | Model 6 |
| --- | --- | --- |
| Gini coefficient | 0.040** | -0.002 |
|  | [0.016, 0.065] | [-0.010, 0.005] |
| Gender |  |  |
| Female | Ref. | Ref. |
| Male | 0.021** | -0.026** |
|  | [0.007, 0.036] | [-0.042, -0.010] |
| Age | 0.002*** | 0.002*** |
|  | [0.001, 0.002] | [0.002, 0.003] |
| Education |  |  |
| Primary or less | Ref. | Ref. |
| Secondary | -0.023* | -0.017 |
|  | [-0.043, -0.003] | [-0.040, 0.006] |
| Tertiary | -0.057*** | -0.056*** |
|  | [-0.079, -0.034] | [-0.080, -0.031] |
| Marital status |  |  |
| Married, civil partnership | Ref. | Ref. |
| Never married, single | -0.016 | -0.021 |
|  | [-0.037, 0.005] | [-0.043, 0.002] |
| Separated or divorced | 0.002 | -0.018 |
|  | [-0.024, 0.027] | [-0.046, 0.009] |
| Widowed | 0.030 | 0.010 |
|  | [-0.005, 0.064] | [-0.023, 0.042] |
| Employment status |  |  |
| In paid work | Ref. | Ref. |
| Unemployed | 0.027 | 0.002 |
|  | [-0.016, 0.069] | [-0.036, 0.039] |
| Not in labor force | -0.025** | -0.009 |
|  | [-0.042, -0.008] | [-0.028, 0.010] |
| Income |  |  |
| Lowest tertile | Ref. | Ref. |
| Middle tertile | -0.017 | -0.021 |
|  | [-0.039, 0.005] | [-0.043, 0.001] |
| Highest tertile | -0.032** | -0.032** |
|  | [-0.055, -0.008] | [-0.056, -0.009] |
| Income missing | -0.019 | -0.052*** |
|  | [-0.041, 0.003] | [-0.076, -0.028] |
| Household size | 0.006* | 0.010*** |
|  | [0.000, 0.012] | [0.004, 0.016] |
| Chronic illness or disability |  |  |
| No | Ref. | Ref. |
| Yes | 0.095*** | 0.078*** |
|  | [0.079, 0.111] | [0.061, 0.095] |
| GDP per capita | 0.012** | 0.002 |
|  | [0.004, 0.020] | [-0.000, 0.003] |
| Constant | -1.241** | 0.093 |
|  | [-2.029, -0.453] | [-0.202, 0.389] |
| Two-way FE | Yes | Yes |
| Number of countries | 5 | 5 |
| Number of country-years | 10 | 10 |
| Observations | 11,586 | 11,352 |
| Adjusted R-squared | 0.039 | 0.053 |
| *** p<.001, ** p<.01, * p<.05. 95% confidence intervals in brackets. | | |

Table S5. Results of Two-Way Fixed-Effects Linear Probability Models Predicting Obesity Status, with Expanded Country Samples

|  | Model 7 | Model 8 |
| --- | --- | --- |
| Gini coefficient | 0.010* | -0.004 |
|  | [0.001, 0.019] | [-0.011, 0.002] |
| Gender |  |  |
| Female | Ref. | Ref. |
| Male | 0.015* | -0.009 |
|  | [0.003, 0.027] | [-0.021, 0.003] |
| Age | 0.002*** | 0.001*** |
|  | [0.001, 0.002] | [0.001, 0.002] |
| Education |  |  |
| Primary or less | Ref. | Ref. |
| Secondary | -0.023** | -0.021* |
|  | [-0.039, -0.007] | [-0.038, -0.004] |
| Tertiary | -0.057*** | -0.060*** |
|  | [-0.074, -0.039] | [-0.077, -0.042] |
| Marital status |  |  |
| Married, civil partnership | Ref. | Ref. |
| Never married, single | -0.016 | -0.024** |
|  | [-0.034, 0.001] | [-0.041, -0.007] |
| Separated or divorced | -0.008 | -0.013 |
|  | [-0.029, 0.013] | [-0.032, 0.006] |
| Widowed | 0.011 | 0.003 |
|  | [-0.015, 0.038] | [-0.021, 0.028] |
| Employment status |  |  |
| In paid work | Ref. | Ref. |
| Unemployed | 0.028 | 0.002 |
|  | [-0.005, 0.061] | [-0.027, 0.030] |
| Not in labor force | -0.018* | -0.017* |
|  | [-0.032, -0.004] | [-0.031, -0.002] |
| Income |  |  |
| Lowest tertile | Ref. | Ref. |
| Middle tertile | -0.024** | -0.021* |
|  | [-0.042, -0.006] | [-0.037, -0.004] |
| Highest tertile | -0.035*** | -0.038*** |
|  | [-0.054, -0.015] | [-0.056, -0.020] |
| Income missing | -0.030** | -0.047*** |
|  | [-0.048, -0.012] | [-0.065, -0.029] |
| Household size | 0.005* | 0.008*** |
|  | [0.000, 0.010] | [0.004, 0.013] |
| Chronic illness or disability |  |  |
| No | Ref. | Ref. |
| Yes | 0.097*** | 0.082*** |
|  | [0.084, 0.110] | [0.070, 0.095] |
| GDP per capita | 0.002 | -0.000 |
|  | [-0.001, 0.006] | [-0.001, 0.001] |
| Constant | -0.239 | 0.327** |
|  | [-0.520, 0.043] | [0.092, 0.562] |
| Two-way FE | Yes | Yes |
| Number of countries | 5 | 5 |
| Number of country-years | 10 | 10 |
| Observations | 11,586 | 11,352 |
| Adjusted R-squared | 0.038 | 0.050 |
| *** p<.001, ** p<.01, * p<.05. 95% confidence intervals in brackets. | | |

Table S6. Results of Two-Way Fixed-Effects Linear Probability Models Predicting Obesity Status, with Multiple Imputation

|  | Model 9 | Model 10 |
| --- | --- | --- |
| Gini coefficient | 0.021*** | -0.007 |
|  | [0.009, 0.033] | [-0.014, 0.000] |
| Gender |  |  |
| Female | Ref. | Ref. |
| Male | 0.017* | -0.018* |
|  | [0.003, 0.030] | [-0.032, -0.003] |
| Age | 0.002*** | 0.002*** |
|  | [0.001, 0.002] | [0.001, 0.002] |
| Education |  |  |
| Primary or less | Ref. | Ref. |
| Secondary | -0.019* | -0.017 |
|  | [-0.037, -0.001] | [-0.039, 0.005] |
| Tertiary | -0.054*** | -0.061*** |
|  | [-0.075, -0.034] | [-0.085, -0.037] |
| Marital status |  |  |
| Married, civil partnership | Ref. | Ref. |
| Never married, single | -0.017 | -0.032** |
|  | [-0.036, 0.003] | [-0.053, -0.011] |
| Separated or divorced | -0.008 | -0.019 |
|  | [-0.032, 0.015] | [-0.043, 0.005] |
| Widowed | 0.020 | -0.003 |
|  | [-0.010, 0.051] | [-0.034, 0.027] |
| Employment status |  |  |
| In paid work | Ref. | Ref. |
| Unemployed | 0.020 | -0.004 |
|  | [-0.017, 0.057] | [-0.039, 0.030] |
| Not in labor force | -0.015 | -0.024** |
|  | [-0.031, 0.001] | [-0.042, -0.007] |
| Income |  |  |
| Lowest tertile | Ref. | Ref. |
| Middle tertile | -0.010 | -0.017 |
|  | [-0.028, 0.007] | [-0.038, 0.003] |
| Highest tertile | -0.025* | -0.029* |
|  | [-0.049, -0.002] | [-0.054, -0.005] |
| Household size | 0.007* | 0.008** |
|  | [0.002, 0.012] | [0.003, 0.014] |
| Chronic illness or disability |  |  |
| No | Ref. | Ref. |
| Yes | 0.098*** | 0.089*** |
|  | [0.083, 0.113] | [0.073, 0.104] |
| GDP per capita | 0.007** | 0.000 |
|  | [0.002, 0.012] | [-0.001, 0.001] |
| Constant | -0.659** | 0.378** |
|  | [-1.065, -0.252] | [0.130, 0.627] |
| Two-way FE | Yes | Yes |
| Number of countries | 5 | 5 |
| Number of country-years | 10 | 10 |
| Observations | 11,586 | 11,352 |
| *** p<.001, ** p<.01, * p<.05. 95% confidence intervals in brackets. | | |
